# Zika Virus Dissemination from the Midgut of *Aedes aegypti* is Facilitated by Bloodmeal-Mediated Structural Modification of the Midgut Basal Lamina

**DOI:** 10.3390/v11111056

**Published:** 2019-11-14

**Authors:** Yingjun Cui, DeAna G. Grant, Jingyi Lin, Xiudao Yu, Alexander W. E. Franz

**Affiliations:** 1 Department of Veterinary Pathobiology, University of Missouri, Columbia, MO 65211, USA; cuiyingj@missouri.edu (Y.C.); LinJin@missouri.edu; (J.L.); yuxiadao@163.com (X.Y.); 2 Electron Microscopy Core Facility, University of Missouri, Columbia, MO 65211, USA; GrantDe@missouri.edu

**Keywords:** Zika virus, dengue 4 virus, mosquito, midgut, dissemination, basal lamina, nanoparticles, consecutive bloodmeals, electron microscopy, immunofluorescence assay

## Abstract

The arboviral disease cycle requires that key tissues in the arthropod vector become persistently infected with the virus. The midgut is the first organ in the mosquito that needs to be productively infected with an orally acquired virus. Following midgut infection, the virus then disseminates to secondary tissues including the salivary glands. Once these are productively infected, the mosquito is able to transmit the virus to a vertebrate host. Recently, we described the midgut dissemination pattern for chikungunya virus in *Aedes aegypti*. Here we assess the dissemination pattern in the same mosquito species for Zika virus (ZIKV), a human pathogenic virus belonging to the *Flaviviridae*. ZIKV infection of secondary tissues, indicative of dissemination from the midgut, was not observed before 72 h post infectious bloodmeal (pibm). Virion accumulation at the midgut basal lamina (BL) was only sporadic, although at 96–120 h pibm, virions were frequently observed between strands of the BL indicative of their dissemination. Our data suggest that ZIKV dissemination from the mosquito midgut occurs after digestion of the bloodmeal. Using gold-nanoparticles of 5 nm and 50 nm size, we show that meal ingestion leads to severe midgut tissue distention, causing the mesh width of the BL to remain enlarged after complete digestion of the meal. This could explain how ZIKV can exit the midgut via the BL after bloodmeal digestion. Ingestion of a subsequent, non-infectious bloodmeal five days after acquisition of an initial, dengue 4 virus containing bloodmeal resulted in an increased number of virions present in the midgut epithelium adjacent to the BL. Thus, subsequent bloodmeal ingestion by an infected mosquito may primarily stimulate de novo synthesis of virions leading to increased viral titers in the vector.

## 1. Introduction

Zika virus (*Flaviviridae*; *Flavivirus*; ZIKV) is a human pathogenic arthropod-borne virus (arbovirus) transmitted by mosquitoes. Originally discovered in 1947 in Uganda, the virus was introduced to Brazil in 2014 due to human activity [1,2,3,4]. In the following years, ZIKV caused major outbreaks among humans in South America, Central America, and in the Caribbean (reviewed in [5]). Typical disease symptoms include febrile illness, rash, headache, and arthritis [3]. Under certain circumstances, a fetus can develop severe neurological damage such as microcephaly when the mother has been infected with the virus [6,7,8]. The rapid ZIKV outbreaks in the Western Hemisphere were fueled by the ubiquitous presence of the mosquito vectors, *Aedes aegypti* and *Ae. albopictus*, both transmitting strains of the virus in urban disease cycles, albeit with varying levels of efficiency [9,10,11,12].

An arthropod vector needs to become persistently infected with a virus in order to transmit the virus to a vertebrate host [13]. A mosquito vector such as *Ae. aegypti* acquires an arbovirus, i.e., ZIKV, along with a viremic bloodmeal from a vertebrate host (reviewed in [14]). The bloodmeal enters the lumen of the mosquito midgut. Within a few hours (before the peritrophic matrix is formed) the virus has to enter midgut epithelial cells via receptor-mediated endocytosis. Flavivirus RNA replication and de novo synthesis of virions take place at the endoplasmic reticulum (ER) [15,16]. De novo synthesized virions, having a diameter of 40–50 nm [17,18], are then transported to the epithelial cells’ basal labyrinth in close proximity to the midgut basal lamina (BL). The BL is produced and secreted by the epithelial cells and constitutes a multi-stranded network of the extracellular matrix components lining the midgut epithelium [19,20]. Earlier studies indicated that flaviviruses, similar to alphaviruses [21] exit the midgut by traversing the BL to disseminate to secondary tissues [22,23]. The secondary tissue tropism within the mosquito vector has been described in detail for the related flavivirus, dengue 2 virus (DENV2) [24]. Following its exit from the mosquito midgut, DENV2 infects hemocytes, fat body, nerve tissue, and eventually the mosquito’s salivary glands. Once these are infected and virus gets released into the salivary ducts during salivation, the female mosquito is able to transmit the virus to vertebrate hosts for the remainder of her life.

Bloodmeal ingestion and digestion by the female mosquito strongly effect the structure and morphology of her midgut [21,25,26,27]. Most obvious is the distension of the posterior midgut, up to 20-fold. This distension causes a reduction in the density of microvilli and the stretching of the basal lamina, which appears now straightened. Within the initial 10–12 h post-bloodmeal (pbm), the basal labyrinth becomes enlarged. The peritrophic matrix is evident in the posterior region of the midgut. Around 20 h pbm, lipid inclusions and glycogen deposits become visible in the gut epithelium near the basal labyrinth. The time period from 36 h pbm onwards indicates the ending of the digestive cycle with lipid inclusions and glycogen deposits starting to disappear from the gut epithelium. By 72 h pbm, blood digestion has ended; however, the morphology of the posterior midgut does not entirely regain its original appearance. For example, cells that were joined before bloodmeal ingestion are now disconnected at their basal portion.

The basic components of the BL are type IV collagen, laminin, nidogen/entactin and proteoglycans of the perlecan type [28]. As described in an ultrastructural analysis by Reinhardt and Hecker [29], the midgut BL of female *Ae. aegypti* forms a three-dimensional structure of hexagonal/cuboidal symmetry. Specifically, the BL consists of several stacked layers and each layer exhibits a grid-like superstructure. The width of the mesh formed by the grid-like structure of each BL layer was around 30 nm on average when analyzing the midguts of sugar-fed mosquitoes [29]. The mesh width increased to around 48 nm directly after bloodmeal ingestion, and five days later, when the bloodmeal had been completely digested, the average mesh width became reduced (35 nm), however was still significantly larger than that of sugar-fed mosquitoes [29]. The barrier character of the midgut BL became obvious for viruses when chikungunya virus (*Togaviridae*; *Alphavirus*; CHIKV) was intrathoracically injected into sugar-fed and blood-fed *Ae. aegypti* (strain: Higgs’ White Eye, HWE) females [25]. In seven-day old sugar-fed females (which had never received a bloodmeal before), injected virions, 60–70 nm in diameter [30,31], lined up at the basal side of the BL and seemed unable to traverse it [25]. However, injection of CHIKV into females that had ingested a bloodmeal resulted in the presence and replication of the virus inside the midgut epithelium. Scanning electron microscopy (SEM) imaging showed that the outer surface of midguts obtained from artificially blood-fed females was severely ripped and distorted near the midgut-surrounding muscles, whereas the surface structure of midguts obtained from sugar-fed females looked intact [21,25]. In addition, we showed that substituting blood in the artificial meal for protein (based on bovine serum albumin [BSA]) or PBS (phosphate buffered saline, no nutrients) had no inhibitory effect on CHIKV dissemination [25]. These combined observations led us to conclude that the presence of a (blood)meal in the midgut is temporally facilitating the dissemination of CHIKV from the organ as it causes the midgut BL to be overly distended thereby increasing its pore size exclusion limit.

Flaviviruses employ replication and virion assembly strategies that differ from those of alphaviruses (reviewed in [32,33,34]). In a mosquito cell, flavivirus replication takes place within replication complexes residing within double-membrane vesicles, which are located at the membrane of the rough ER [15]. Flaviviruses express their structural and non-structural proteins from the same RNA molecule [32,33]. De novo synthesized viral RNA forms a complex with the transmembrane capsid (C) protein. The capsid protein-RNA complex receives a lipid bilayer envelope originating from the ER containing the structural proteins envelope (E) and pre-membrane (prM). This results in the formation of an immature fusion-incompetent virion, which is budding into the ER lumen. When transported through the trans-Golgi network, E and prM undergo conformational changes forming a mature virion with prM molecules now covering the fusion loops of E. Via furin cleavage, prM is eventually cleaved into pr and M. Alphaviruses, by contrast, express their nonstructural and structural proteins from different viral RNA subunits and regulate their plus/minus strand RNA and protein synthesis in a temporal manner enabling them to quickly replicate to high titers [32]. Virion assembly of alphaviruses within the infected cell involves a two-step assembly process [34,35]. Viral RNA replication is controlled by the non-structural proteins (nsP1-nsP4) and takes place within virus-induced replication factories consisting of spherules located at the plasma membrane and within large cytopathic vacuoles [36]. Translation of the subgenomic RNA produces the structural polyprotein containing CP (capsid protein), E3, E2, 6K, and E1 [34,35]. CP is cleaved from the polyprotein and freely released into the cytoplasm to form the nucleocapsid together with de novo synthesized viral RNA released from the spherules. The glycoprotein precursors PE2 (including E2 and E3) and E1 are translocated across the ER and then processed and transported though the trans-Golgi network to the plasma membrane. There, the nucleocapsid, having bypassed the trans-Golgi network, associates with the glycoproteins, resulting in the assembly of mature virions, each now possessing 80 spikes consisting of trimers of E2 and E1 heterodimers [32,34,36].

Generally, alphaviruses build up higher titers in a shorter time period than flaviviruses do in the same mosquito vector [23,37]. This prompted us to investigate whether the different infection dynamics between flaviviruses and alphaviruses are also leading to different midgut dissemination patterns for the two virus groups. Alternatively, the mechanics underlying distention and reconfiguration of the mosquito midgut/BL structure during bloodmeal digestion could perhaps dictate a common dissemination pattern for both virus groups. Here, we investigate the dissemination pattern of the flavivirus, ZIKV, from the mosquito midgut and discuss whether it differs from that previously described for the alphavirus, CHIKV [21,38]. In an in vivo growth curve, we revealed the time point when orally acquired ZIKV was detectable outside the mosquito midgut. In parallel, we observed infection patterns for the virus in situ using immunofluorescence assays and at the ultrastructural level via transmission electron microscopy (TEM). Furthermore, using a mixture of 5 nm and 50 nm gold nanoparticles, we show how meal ingestion into the midgut changed the size exclusion limit of the BL and discuss its relevance for the midgut dissemination of ZIKV. The effect of multiple bloodmeal ingestions by the mosquito on the intensity of infection was assessed for the flavivirus, dengue 4 virus (DENV4), at the ultrastructural level.

## 2. Materials and Methods

### 2.1. Mosquitoes and Viruses

The eye pigment deficient Higgs’ White Eye (HWE) strain of *Ae. aegypti* [39] was maintained in an insectary under a 12 h light/12 h dark cycle and a temperature and humidity regime of 28 °C and 80%, respectively. Larvae were reared at a moderate density (~100 larvae per shoe box size hatch pan and ~3 cm distilled water level) and fed with tropical fish food (Tetramin, Melle, Germany). One hundred emerged females and around 10 males were placed in a 64 oz. (~1 L) ice cream carton, which was covered with netting. Mosquitoes were supplied ad libitum with raisins and water. One-week post-emergence, females were used for experiments involving ZIKV, DENV4, or gold-nanoparticles.

ZIKV strain I-44 (GenBank #: KX856011), which was isolated in 2016 from mosquitoes captured in Chiapas State of Mexico was used in this study [10,40]. The virus was propagated for four days (until use in artificial blood-feeding experiments) in confluent Vero cells (ATCC #: CCL-81) at a multiplicity of infection (MOI) of 0.01. Vero cells were cultured in complete Dulbecco’s Modified Eagle Medium (DMEM) supplemented with 7% fetal bovine serum (FBS). DENV4 strain H241 (GenBank #: KR011349) was isolated from a human patient in the Philippines in 1956 and represents the prototype for DENV serotype 4 [41,42]. *Ae. albopictus* C6/36 cells were cultivated at 28 °C without CO_2_ supplement in Leibovitz L-15 medium supplemented with 7% FBS until 80%–90% confluency. Cells were then infected with DENV4 at a MOI of 0.001. Following virus inoculation, the cell culture medium was changed for fresh L-15 medium now containing 2% FBS and 1% non-essential amino acids (100x) (Corning Inc., Corning, NY, USA). Following 4–5 days of cultivation, the cell culture supernatant was harvested and used in artificial bloodmeals for oral infection of mosquitoes. All research work involving ZIKV and DENV4 was performed in the biosafety level 3 (BSL3) Virology Suite of the Laboratory for Infectious Disease Research (LIDR) at the University of Missouri.

### 2.2. ZIKV or DENV4 Infection of Mosquitoes via Artificial Bloodmeals

Twenty-four hours before blood-feeding, HWE females were deprived of their raisin food source and their water cups were removed 2–3 h prior to blood-feeding. ZIKV-containing Vero cell culture supernatant (at 96 h post-infection pi)) or DENV4-containing C6/36 cell culture supernatant (at 96–120 h pi) was mixed at a 1:1 ratio with defibrinated sheep blood (Colorado Serum Company, Denver, CO, USA) and 10 mM ATP. The artificial bloodmeal was pipetted into glass feeders (2 mL volume/feeder, one feeder/carton), which were covered with a hog gut membrane to be exposed to the mosquitoes in the carton for probing. The glass feeders were connected to a water jacket to maintain a temperature of 37 °C. Mosquitoes were allowed to feed for 1 h before fully engorged females were selected and maintained for further analysis in an environmental growth chamber at 28 °C and 80% relative humidity.

### 2.3. Plaque Assays for the Detection of ZIKV in Individual Mosquito Tissues

At 24, 48, 72, 96, 120, and 168 h post-infectious bloodmeal (pibm), midguts of blood-fed mosquitoes were dissected and separately maintained from the remaining body (carcass). To assess viral titers in individual tissues, midguts and carcasses were placed individually into 1.5 mL micro-centrifuge tubes containing 0.5–1.0 mL DMEM supplemented with 7% FBS, 5% HEPES and ground with a micro pestle before being filtrated using 0.22 µm Supor Membrane syringe filters (Pall Life Sciences, East Hills, NY, USA). Filtrated samples were 10-fold diluted and 150 µl of sample volume for each dilution step was used to inoculate ~90% confluent Vero cells seeded into 24-well plates. After a 1 h incubation period at 37 °C, inoculated Vero cells were overlaid with a 1% agarose-nutrient mixture containing 10% M199 (10x), 7% FBS, 0.5% MEM vitamin solution (100x), 0.5% MEM non-essential amino acid solution (100x), and 0.003% sodium bicarbonate solution (Gibco, ThermoFisher, Waltham, MA, USA). Following a four-day incubation period at 37 °C under 5% CO_2_ supplement, agarose containing plates were fixed with 10% formalin for 5 h. Thereafter, agarose ‘plugs’ were carefully removed without disturbing the Vero cell layer and cells were briefly stained with 0.2% crystal violet solution to visualize plaques. Plaques of each well were counted under a light microscope to calculate viral titers as plaque-forming units/mL (PFU/mL).

### 2.4. Detection of Flaviviral Antigen in Individual Mosquito Tissues by Immunofluorescence Assay (IFA)

At 24, 48, 72, 96, 120, and 168 h pibm, midguts were dissected and fixed in 4% para-formaldehyde dissolved in 1x PBS. Fixed midguts were permeabilized using PBS-T (1x PBS containing 0.2% Triton X-100 and 1% BSA), before a 1 h incubation at room temperature (RT) with the primary monoclonal antibody Anti-Flavivirus Group Antigen D1-4G2-4-15 (ATCC #: VR-1852), diluted 1:500 in PBS-T. Following several washes with PBS-T, the secondary monoclonal antibody (goat anti-mouse IgG labeled with Alexa Fluor 594, Abcam # ab150120) was applied for 1 h at RT at a 1:500 dilution in PBS-T together with Alexa Fluor Phalloidin 488 (Invitrogen, Carlsbad, CA, USA) diluted 1:1000. Cell nuclei were stained with 1 µg/mL DAPI (Invitrogen) for 10 min. Midguts were then washed three times with PBS before being mounted on six-well printed slides, using Fluoromount G mounting medium (Electron Microcopy Sciences, Hatfield, PA, USA). Samples were viewed under an inverted spectral confocal microscope (TCP SP8 MP, Leica Microsystems, Wetzlar, Germany) at the Molecular Cytology Core of the University of Missouri.

### 2.5. Detection of ZIKV and DENV4 in Midgut Tissue by TEM

Midguts obtained from ZIKV infected (at 32, 72, 80, 96, and 120 h pibm) and DENV4 infected (at 144 h pibm) females were fixed in 100 mM sodium cacodylate buffer, pH 7.35 (Sigma Aldrich, St. Louis, MO, USA) supplemented with 2% paraformaldehyde and 2% glutaraldehyde. Sample embedding, ultrathin-sectioning, and TEM work were performed at the Electron Microscopy Core of the University of Missouri. Specimens were embedded in HistoGel (Thermo Scientific, Kalamazoo, MI, USA) before being rinsed with 100 mM sodium cacodylate buffer containing 130 mM sucrose. Secondary fixation was conducted in a Pelco Biowave (Ted Pella, Redding, CA, USA) using 100 mM sodium cacodylate buffer supplemented with 1% osmium tetroxide. Following a 1 h incubation at 4 °C, samples were en bloc stained over-night at 4 °C using 1% aqueous uranyl acetate. A graded dehydration series (100 Watts for 40 s per exchange) was performed in a Pelco Biowave, in which ethanol was initially chosen, followed by transition to acetone before dehydrated specimens were finally infiltrated with EPON resin (at 250 Watts for 3 min) and polymerized at 60 °C overnight. Embedded samples were ultrathin-sectioned (85 nm) using an ultra-microtome (Ultracut UCT, EM UC7, Leica Microsystems, Wetzlar, Germany) equipped with a diamond knife (Diatome, Hatfield, PA, USA). TEM images were acquired at 80 kV with a JEOL JEM 1400 transmission electron microscope (JEOL, Peabody, MA, USA), which was connected to a Gatan Ultrascan 1000 CCD camera (Gatan, Pleasanton, CA, USA).

### 2.6. Treatment of Dissected Midguts with Gold-Nanoparticles

Seven days-old HWE mosquitoes were deprived of food for one day and water for 4 h before receiving a proteinmeal consisting of 20% BSA using artificial glass feeders as described above. Fully engorged mosquitoes were selected and their midguts dissected at 24, 48, 60, 72, 96, 120, and 168 h post-feeding (pf). Dissected midguts were soaked for 2 h in a suspension consisting of 5 nm FITC labeled gold-nanoparticles (1.99 × 10^9^ nanoparticles/mL) and 50 nm Cy3 labeled gold-nanoparticles (1.99 × 10^9^ nanoparticles/mL) (Nanopartz, Loveland, CO, USA). Midguts were then washed three times with 1x PBS before being stained with 1 µg/mL DAPI for 10 min. Following several washes with PBS, midguts were mounted on six-well printed slides and viewed under an inverted spectral confocal microscope (TCP SP8 MP, Leica Microsystems). A 3D-image was obtained from a midgut sample of a proteinmeal-fed female at 72 h pf. 3D-image reconstruction along the x, y, and z axes was performed using the LAS X 3D imaging software (Leica, Wetzlar, Germany).

### 2.7. Scanning Electron Microscopy (SEM) and Scanning Transmission Electron Microscopy (STEM) for the Detection of Gold-Nanoparticles in Midgut Samples

The initial SEM sample preparation steps followed the protocol for TEM sample preparation as described above. Following the graded dehydration series, samples were dried using the Tousimis Autosamdri 815 critical point dryer (Tousimis, Rockville, MD, USA), and then sputter-coated with 10 nm of platinum using the EMS 150T-ES Sputter Coater. Images were acquired with a FEI Quanta 600F environmental scanning electron microscope (FEI, Hillsboro, OR, USA). The STEM sample preparation procedure was similar to the TEM protocol as described above. Following the soaking of the midguts in the gold-nanoparticle suspension, samples were collected and processed for TEM as described. Scanning transmission electron microscopy of the TEM preparations (STEM) was performed in high angle annular dark field (HAADF) image mode on a ThermoFisher Tecnai F30 Twin 300 kV TEM operated at 200 kV. The intensity of STEM-HAADF imaging was proportional to the atomic number Z1.7, enabling gold-nanoparticles to be identified as bright particles in the sample.

## 3. Results

### 3.1. ZIKV Disseminates from the Mosquito Midgut After Bloodmeal Digestion

**Figure 1:**
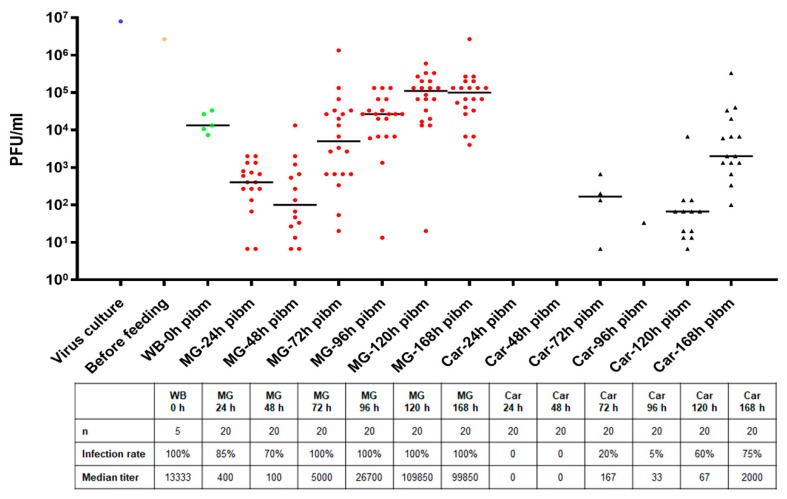
Midgut infection and dissemination patterns of Zika virus (ZIKV) I-44 in individual tissues following oral acquisition of the virus by *Ae. aegypti* (strain: Higgs’ White Eye (HWE)). The virus titer in the bloodmeal was 1.3 × 10^6^ PFU/mL. ZIKV titers in individual midguts (MG; *n* = 20/time point) and carcasses (Car; *n* = 20/time point) were assessed by plaque assays in Vero cells at 0, 24, 48, 72, 96, 120, and 168 h post-infectious bloodmeal (pibm). Bars indicate median values. Only those individual tissues that were infected are presented in the graph. Statistical analysis was performed using the Mann–Whitney U-test to analyze the intensities of infection. WB = whole body.

Orally acquired ZIKV I-44 productively infected *Ae. aegypti* females within a seven-day period. When feeding for 1 h on an artificial bloodmeal containing 1.3 × 10^6^ PFU/mL virus, each female ingested the virus with a median concentration of ~13,300 PFU/mL (Figure 1). In individual midguts, an eclipse phase was observed until 48 h pibm causing a decrease of the median midgut virus titer to just 100 PFU/mL. Thereafter, de novo synthesis of ZIKV increased steeply to a median value of 5000 PFU/mL at 72 h pibm. By 120 h pibm, individual midgut virus titers reached a median peak value of ~100,000 PFU/mL thereby exceeding the median input virus titer by 10-fold. Midgut infection rates during the time period varied between 70% and 100%. Carcass infection rates, by contrast, did not exceed 50% before 120 h pibm and at 168 h pibm, when 75% of carcasses were infected, median virus titers were just 2000 PFU/mL. Importantly, ZIKV was undetectable in carcass tissue before 72 h pibm indicative of a relatively prolonged period before the virus exited the midgut and productively infected secondary tissues. By 72 h pibm, the ingested bloodmeal was completely digested although occasionally, a tiny remainder of blood was still visible in a dissected midgut. Midguts of blood-fed mosquitoes were typically slightly more distended at 72 h pibm than midguts of sugar-fed females [21].

### 3.2. ZIKV Infection Foci Progressively Increase in the Midgut Epithelium over Time

**Figure 2:**
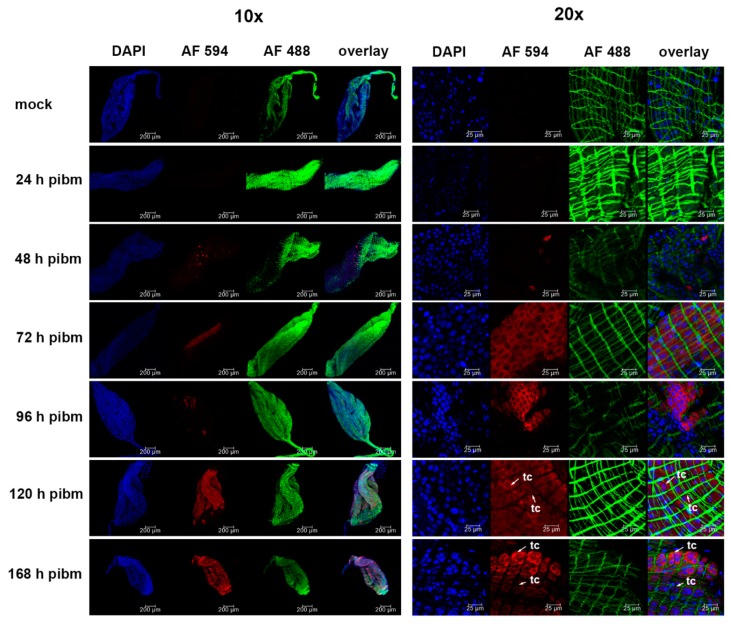
In situ detection of ZIKV antigen in midguts of *Ae. aegypti* by immunofluorescence assay. Midguts dissected from females at 24, 48, 72, 96, 120, and 168 h pibm (ZIKV titer in the bloodmeal: 2.7 × 10^6^ PFU/mL) were incubated with flavivirus-specific monoclonal antibody (IgG) 4G2 followed by incubation with secondary anti-mouse monoclonal antibody (IgG) labeled with Alexa Fluor (AF) 594 (red). Green: staining of actin filaments with Alexa Fluor (AF) Phalloidin 488; blue: staining of nuclei with DAPI. Images of two different magnifications, 10x and 20x, are shown. Mock: non-infected midgut sample incubated with monoclonal antibody (IgG) 4G2 followed by incubation with anti-mouse monoclonal antibody (IgG). Images were captured using an inverted spectral confocal microscope (TCP SP8 MP, Leica Microsystems). tc = tracheal cell.

Using monoclonal antibody 4G2 in IFA, ZIKV antigen was detected in the midgut epithelium of *Ae. aegypti* from 48 h pibm onwards but not at 24 h pibm or earlier (Figure 2). At 48 h pibm, just individual, isolated midgut cells (<50 cells/midgut) showed the presence of viral antigen. From 72 h onwards, however, infection foci comprised of dense clusters of infected cells became apparent. These infection foci became enlarged over time until they encompassed most of the midgut tissue at 120 or 168 h pibm. Thus, the immunofluorescence studies reflected the results of our ZIKV growth curve in individual midguts (Figure 1) showing that de novo synthesis of the virus occurred from around 48 h pibm onwards with maximal titers being reached at 120–168 h pibm. Importantly, the in situ detection study confirmed that ZIKV did not infect midgut associated tracheal cells or muscle tissue within the seven-day observation period. As shown in Figure 2, there was no co-localization apparent between viral antigen and muscle fibers or tracheal cells. Thus, ZIKV disseminates from the midgut epithelium to secondary tissues by traversing the midgut BL and not via midgut associated tracheal cells.

### 3.3. ZIKV Accumulates at the Midgut BL at a Relatively Low Density when Disseminating from the Organ After Bloodmeal Digestion

**Figure 3:**
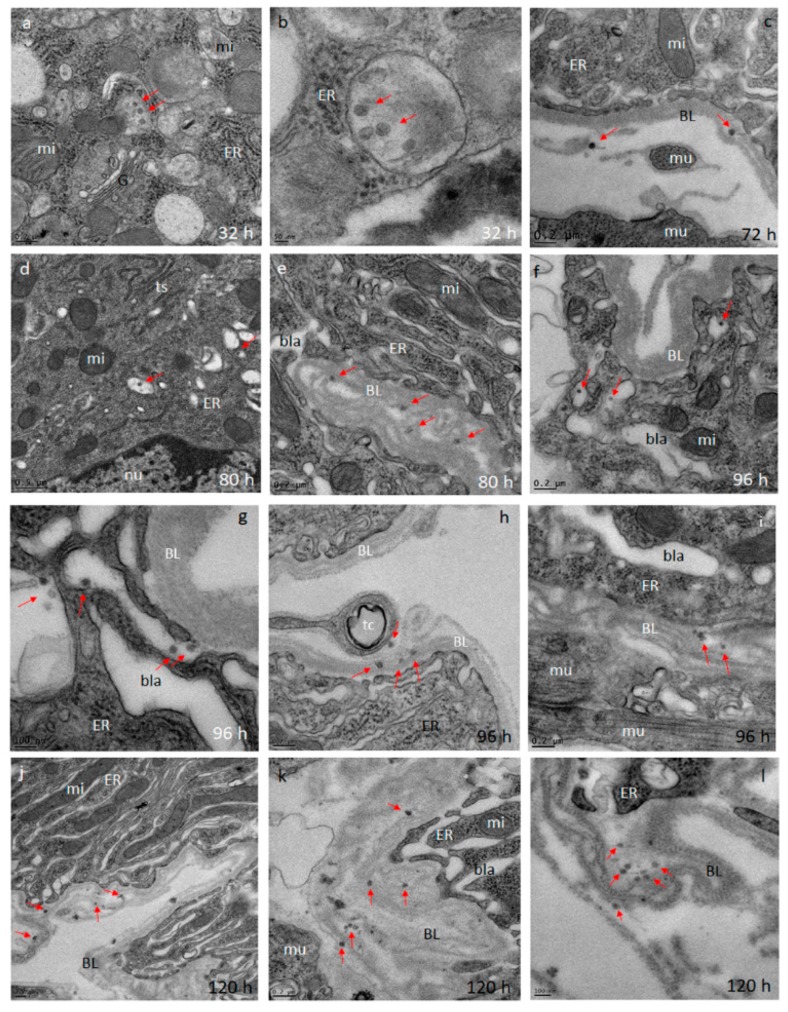
Ultrastructural (transmission electron microscopy (TEM)) imaging of midgut cross-sections obtained from female *Ae. aegypti*, which had been orally infected with ZIKV I-44 (titer in the bloodmeal: 1.3 × 10^6^ PFU/mL). (**a,b**) Cross sections through the midgut epithelium of *Ae. aegypti* HWE females at 32 h pibm. In a few epithelial cells, Zika (ZIK) virions (exemplified by red arrows) are visible within membranous/vesicular compartments. Virions were not detected at the midgut basal lamina (BL). (**c**) At 72 h pibm, ZIK virions appear at the midgut BL nearby muscle tissue. This indicates the beginning of virus dissemination from the midgut by traversing the BL. (**d,e**) Eight hours later, virions were occasionally observed in compartments shown in (**a,b**). Further, virions accumulated within strands of the midgut BL indicative of their dissemination from the midgut. (**f–i**) This trend continued at 96 h pibm, with virions also accumulating within the epithelial cell’s basal labyrinth in close proximity to the BL. (**j–l**) At 120 h pibm, a stronger accumulation of ZIK virions within strands of the midgut BL was visible. At least three different midgut samples per time point were analyzed. Images were generated using a JEOL JEM 1400 transmission electron microscope. Magnifications ranged from 2500 to 10,000. BL = basal lamina; bla = basal labyrinth; ER = endoplasmic reticulum; G = trans-Golgi network; mu = muscle tissue; mi = mitochondrion; nu = nucleus; tc = tracheal cell; ts = tubular structure.

Using TEM imaging, we observed the presence of Zika (ZIK) virions in midgut tissue at 32, 72, 80, 96, and 120 h pibm (Figure 3). At 32 pibm (*n* = 3–5 midgut samples), clusters of de novo synthesized virions were found within vesicular structures located in the ER region, which was not in close proximity to the BL (Figure 3a,b). From 72 h pibm onwards, isolated groups of ZIK virions (<10 virions in a group) were detected in the basal labyrinth of the epithelial cell in close proximity to the BL and within strands of the BL, suggesting dissemination from the midgut (Figure 3c–i). At 120 h pibm, groups of <20 virions were found within strands of the BL, while there were hardly any virions detected in the adjacent basal labyrinth (Figure 3j–l). Neither midgut associated tracheal cells nor muscle tissue were found to be infected with ZIKV during the 120 h time course. In line with the results of our ZIKV in vivo growth curve (Figure 1), the ultrastructural observations here suggest that ZIKV dissemination from the midgut occurs from around 72 h pibm onwards. Typically, the ingested bloodmeal was completely digested by then.

### 3.4. Proteinmeal Ingestion Increases the Mesh Width of the Midgut BL

**Figure 4:**
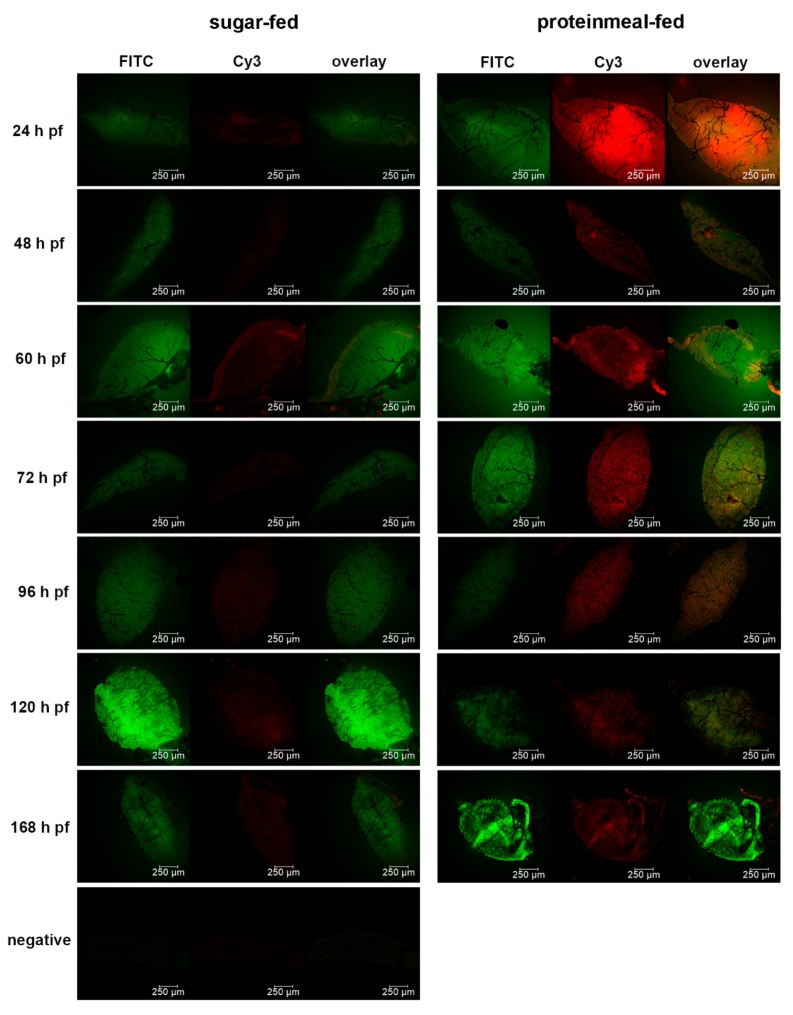
Detection of 5 nm and 50 nm gold-nanoparticles in midguts obtained from sugar-fed and proteinmeal-fed (20% BSA solution) mosquitoes at various time points post-feeding (pf) by fluorescence microscopy. Dissected midguts were soaked for 2 h in a suspension containing 5 nm (1.99 × 10^9^ nanoparticles/mL, labeled with FITC) and 50 nm (1.99 × 10^9^ nanoparticles/mL, labeled with Cy3) gold-nanoparticles before mounting and viewing. Midgut samples were viewed under an inverted spectral confocal microscope (TCP SP8 MP, Leica Microsystems) thereby maintaining the same imaging parameters for each sample.

**Figure 5:**
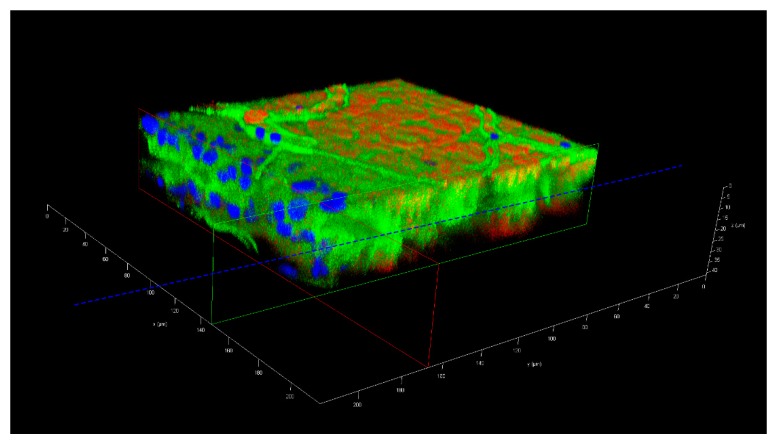
Three-dimensional confocal microscopy image of a midgut preparation, which had been soaked for 2 h in a suspension containing 5 nm (1.99 × 10^9^ nanoparticles/mL, labeled with FITC) and 50 nm (1.99 × 10^9^ nanoparticles/mL, labeled with Cy3) gold-nanoparticles. The midgut was dissected from a female mosquito at 72 h post-oral acquisition of a proteinmeal consisting of a 20% BSA solution. The dashed blue line depicts the zone of the midgut lumen, the horizontal green and red lines the location of the midgut surfaces. The image was captured using an inverted spectral confocal microscope (TCP SP8 MP, Leica Microsystems). Blue = nuclei stained with DAPI; green = FITC labeled 5 nm gold-nanoparticles; red = Cy3 labeled 50 nm gold-nanoparticles. Irregular line structures (green) represent tracheae.

**Figure 6:**
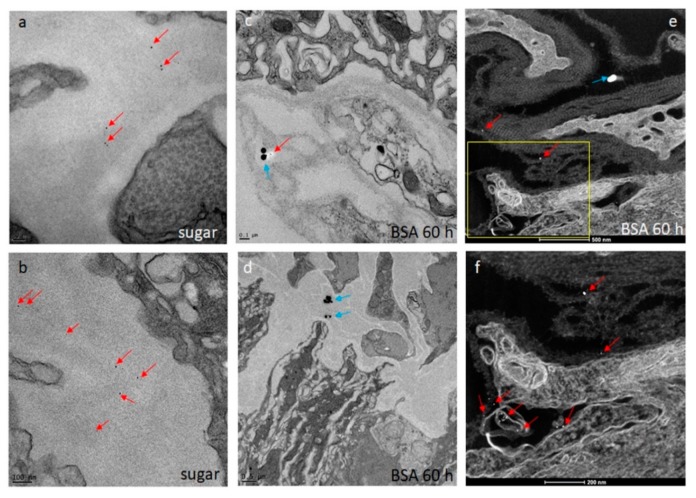
Ultrastructural (TEM) detection of 5 nm gold-nanoparticles (red arrows) and 50 nm gold-nanoparticles (blue arrows) within strands of the midgut BL (**a–d**). Midguts were obtained from sugar-fed (**a,b**) and proteinmeal-fed *Ae. aegypti* (**c–f**; at 60 h pf of a 20% BSA solution). Images were captured using a JEOL JEM 1400 transmission electron microscope. (**e,f**) Detection of 5 nm (red arrows) and 50 nm (blue arrow) gold-nanoparticles associated with the midgut BL by scanning transmission electron microscopy (STEM) to enhance visibility of the nanoparticles. Images were generated using a ThermoFisher Tecnai F30 Twin 300kV TEM/STEM operated at 200 kV in high angle annular dark field (HAADF) image mode.

We wanted to elucidate how meal ingestion by the midgut would affect the pore size exclusion limit of its BL over time. We therefore soaked dissected midguts obtained from proteinmeal-fed (20% BSA solution) and sugar-fed mosquitoes at various time points post-feeding in a suspension containing 5 nm (labeled with FITC, 1.99 × 10^9^ nanoparticles/mL) and 50 nm (labeled with Cy3, 1.99 × 10^9^ nanoparticles/mL) gold-nanoparticles to observe under which conditions each nanoparticle type would be able to penetrate (or adhere) to the midgut BL. In contrast to a sugarmeal, which is deposited into the crop of the female, a proteinmeal is routed to the midgut thereby causing its distention. Similar to bloodmeal ingestion [19,23], the high level of BL distention caused by ingestion of a proteinmeal led to ruptures and tearing of the midgut BL as shown by SEM (Figure S1), indicating that the overall BL structure was severely affected. In contrast, there was only minimal damage occasionally visible on the relaxed surface of midguts obtained from sugar-fed mosquitoes. Since the gold-nanoparticles with 5 nm diameter were labeled with FITC dye and the nanoparticles with 50 nm diameter were labeled with Cy3 dye, their presence or absence could be easily distinguished in situ using fluorescence confocal microscopy. Based on fluorescent FITC signaling, our results showed that 5 nm nanoparticles were absorbed by the BL of midguts obtained from all sugar-fed or proteinmeal-fed females at any time point between 24 h and 168 h pf (Table 1, Figure 4, Figure 5 and Figure 6, Figure S2).

During the 24–168 h time course, 39/150 midgut samples obtained from mosquitoes that had ingested a proteinmeal exhibited Cy3 signaling, indicating that the larger 50 nm gold-nanoparticles associated with the midgut BL in those mosquitoes. However, at 168 h pf, an association between 50 nm nanoparticles and the midgut BL of proteinmeal-fed mosquitoes was not evident. The highest quantity of midguts (11/25) showing the presence of 50 nm nanoparticles was observed at 60 h post-proteinmeal feeding, a time point when the meal was already completely digested (Table 1). Furthermore, 9/134 midgut samples obtained from sugar-fed mosquitoes also exhibited 50 nm nanoparticle-specific Cy3 signaling, although the Cy3 signaling intensity was typically much more subdued in these samples as compared to midguts obtained from proteinmeal-fed mosquitoes (Figure S2).

TEM and STEM imaging confirmed ultrastructurally the association of 5 nm gold-nanoparticles with midguts from sugar-fed and proteinmeal-fed females (Figure 6a–f). However, in only a few instances was complete penetration of the BL with 5 nm gold-nanoparticles observed (Figure 6f), confirming that the BL mesh width of the relaxed midgut must be at least 5 nm wide. Gold-nanoparticles of 50 nm diameter were found to associate with midgut tissue originating from proteinmeal-fed females but they did not completely penetrate the midgut BL (Figure 6c,d). Regardless, in the midguts of sugar-fed mosquitoes, the association between BL and 50 nm nanoparticles was clearly less efficient (Table 1). The results of this experiment suggest that after a single meal ingestion, the mesh width of the midgut BL increases and remains so for a prolonged time period after digestion of the ingested meal. This could explain how ZIK virions, having roughly a similar size to those 50 nm gold-nanoparticles would be able to traverse the midgut BL after bloodmeal digestion.

### 3.5. Ingestion of a Second, Non-Infectious Bloodmeal Increases Virion Accumulation of the Flavivirus, DENV4 at the Midgut BL

**Figure 7:**
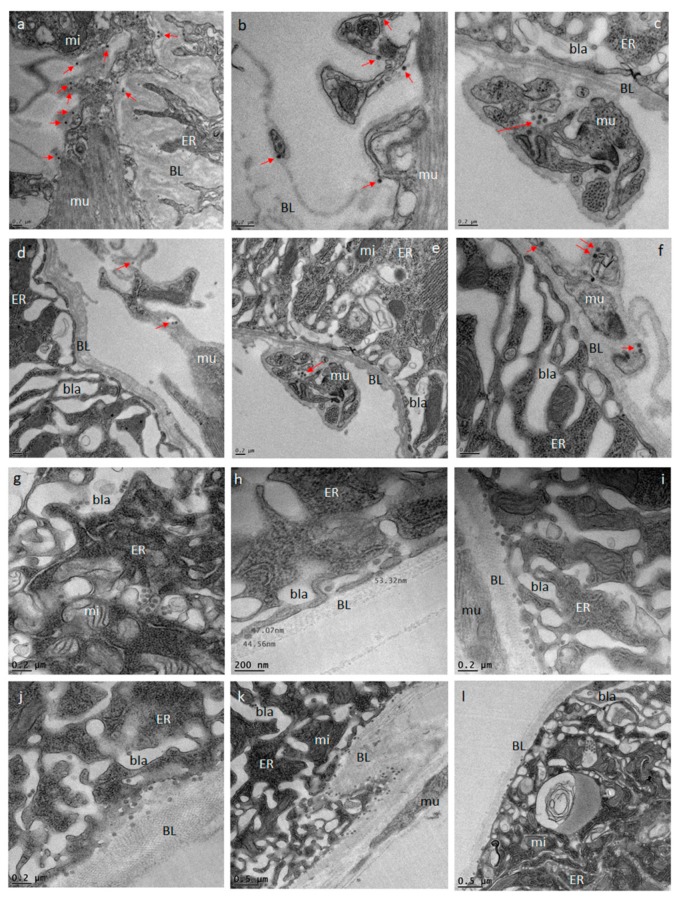
Ultrastructural (TEM) imaging of midgut cross-sections obtained from female *Ae. aegypti*, which had received a single, DENV4 containing bloodmeal in comparison to those that have received a second, non-infectious bloodmeal at 120 h post-oral acquisition of the DENV4 containing bloodmeal. One-week old females received an artificial bloodmeal containing 10^6^ PFU/mL DENV4 (strain H241). At 144 h pibm, midgut tissue cross sections were prepared and analyzed by TEM. (**a–f**) As exemplified by red arrows, relatively low quantities of DEN4 virions were visible within strands of the midgut BL. (**g–l**) When midgut samples were analyzed 24 h after receiving of a second, non-infectious bloodmeal, substantially higher quantities (three to four times as many) of DEN4 virions were visible within membranous inclusions surrounded by the ER as well as in the basal labyrinth and at the epithelial site of the BL. Images were generated using a JEOL JEM 1400 transmission electron microscope. Magnifications ranged from 2500x to 10,000x. BL = basal lamina; bla = basal labyrinth; ER = endoplasmic reticulum; mi = mitochondrion; mu = muscle.

At the ultrastructural level, the infection and dissemination pattern of DENV4 H241 resembled that of ZIKV described above in midguts of mosquitoes that had received a single virus containing bloodmeal (Figure 7a–f). At 144 h pibm, single dengue 4 (DEN4) virions or small groups of fewer than 10 virions were observed at the midgut BL as well as the BL surrounding the muscle tissue. The muscles per se were not found to be infected with the virus. However, when mosquitoes received a second, non-infectious bloodmeal 120 h after the initial, DENV4 containing bloodmeal, DEN4 virions massively congregated and accumulated at the midgut BL at 144 h pibm (Figure 7g–l). Virion density was increased by 3- to 4-fold in comparison to that in the midguts of those mosquitoes, which had only received a single, DENV4 containing bloodmeal. Curiously, only a few virions were visible between the strands of the BL. These observations allow us to conclude that ingestion of second, non-infectious bloodmeal not only leads to repeated midgut BL over-distention, but is also boosting DEN4 virion production in the midgut epithelium.

## 4. Discussion

We investigated the midgut infection and dissemination pattern of ZIKV I-44 from the midgut of *Ae. aegypti* (strain: HWE) by in vivo growth curve analysis, in situ detection of viral antigen over time, and ultrastructurally using TEM with the aim to explain the dissemination mechanism of the virus. In the in vivo growth curve analysis, dissemination of orally acquired ZIKV from the mosquito midgut was not observed before 72 h pibm since there was no infection of secondary tissues prior to that time point. At 72 h pibm, however, the midgut tissue was strongly infected with the virus (median virus titer: 5000 PFU/mL) and the bloodmeal completely digested. Furthermore, median titers in carcasses did not exceed ~170 PFU/mL before 168 h pibm. In our ultrastructural studies, ZIK virions were observed to associate with the midgut BL at 72 h pibm and did not appear in larger quantities between strands of the BL prior to 120–168 h pibm. In situ immunofluorescence assays confirmed our virus growth curve analysis as ZIKV antigen became apparent in the midgut at 48 h pibm, which coincided with the time point of observed viral de novo synthesis in this organ. Maximal presence of viral antigen was observed at 120–168 h pibm, when most parts of the organ looked infected. This matched the time period when the maximal virus titers were observed in the midgut. Furthermore, ZIKV did not infect the midgut associated tracheae or muscles as shown by IFA and TEM. All these observations lead to the conclusion that ZIKV dissemination from the midgut, similar to that of CHIKV, occurs by traversing the midgut BL [21,25,38].

Other than that, the dissemination pattern of ZIKV differed substantially from that of CHIKV in the same strain of *Ae. aegypti*. In a previous study, CHIK virions were already detected outside the mosquito midgut at 24 h pibm, before digestion of the bloodmeal [38]. In the basal labyrinth of the midgut epithelial cells, a strong accumulation of mature virions in the process of traversing the BL, was observed between 24 and 32 h pibm [21]. From 48 h pibm onwards, however, virion accumulation at the BL became less pronounced. We concluded that there was a “window of opportunity” during which large quantities of CHIK virions exited the midgut by traversing the BL during bloodmeal digestion when the midgut tissue was still overly distended. In contrast, as this study suggests, flaviviruses dissemination from the midgut primarily occurs after bloodmeal digestion. As described above, different replication and virion assembly strategies of alpha- and flaviviruses [15,16,32,33,34,35,36] may be the underlying cause for their different infection and dissemination dynamics in *Ae. aegypti*. Furthermore, mature virions of alphaviruses are substantially larger (60–70 nm in diameter) than those of flaviviruses (40–50 nm in diameter) [17,18,30,31]. Consequently, CHIKV may necessitate a more strongly distended midgut BL than ZIKV or DENV4 before being able to disseminate from the mosquito midgut. Bloodmeal ingestion leads to maximal BL distention during which the BL mesh size is significantly increased [29]. Previously, we hypothesized that this bloodmeal induced “over”distention would increase the pore size exclusion limit of the BL sufficiently to enable CHIK virions to pass through [21]. During subsequent bloodmeal digestion, the BL mesh size contracts again [29]. According to our hypothesis, this would then steadily reduce the chance for CHIK virions to be able to traverse the BL. Thus, there would be a relatively narrow time span during which the mesh width of the BL would be just large enough for CHIK virions to pass through, which could explain the previously described “window of opportunity” dissemination pattern for CHIKV during bloodmeal digestion [21]. However, after bloodmeal digestion, the mesh width of the midgut BL does not completely contract all the way to its original (pre-initial bloodmeal) width, which, in an earlier study, measured to be around 30 nm in *Ae. aegypti* [29]. This then could explain why flaviviruses, which are up to ~20 nm smaller in diameter than alphaviruses, would still be able to disseminate from the midgut after digestion of the bloodmeal. To further substantiate the idea, we decided to analyze the permissiveness of the midgut BL before, during, and following bloodmeal digestion using 5 nm and 50 nm size gold-nanoparticles. In agreement with Reinhardt and Hecker [29], our experiments here suggest that following ingestion of an initial proteinmeal, the BL mesh width is enlarged due to midgut tissue distention and remains so for ~70 h after complete digestion of the meal. Whereas 5 nm gold-nanoparticles were consistently absorbed by the midgut BL of sugar-fed and proteinmeal-fed mosquitoes, 50 nm gold-nanoparticles predominantly associated with the BL of midguts obtained from proteinmeal-fed mosquitoes.

In another, earlier ultrastructural analysis, the permeability of the midgut BL of *Culex tarsalis* for various particle sizes was analyzed [43]. It was demonstrated that soaking dissected midguts of sugar-fed *Cx. tarsalis* in suspensions containing lanthanum, horseradish peroxidase, cytochrome C, or colloidal thorium with sizes ranging from <2–8 nm resulted in particle absorption by the midgut BL, with particles occasionally found within the midgut epithelium. Particles of >8 nm in size accumulated at the basal site of the BL but did not traverse it, which is in accordance to our observations using gold-nanoparticles. Unfortunately, the authors did not provide any data showing whether or not particles of >8 nm in size were able to traverse the BL of midguts obtained from blood-fed mosquitoes. However, a statement was provided that there was no difference between sugar-fed and blood-fed mosquitoes, which contradicts previous work and our observations here [29]. Two observations made during our study remain enigmatic: 1) The fact that gold-nanoparticles of either size generally did not completely penetrate or traverse the midgut BL although they were found located between strands of the BL and 2) that in some cases 50 nm nanoparticles were able to associate with the BL of sugar-fed mosquitoes. We speculate that their physical or electrostatic properties including their lack of elasticity/flexibility may have prevented those gold-nanoparticles from completely traversing the BL and entering the midgut epithelium. It may also be that the mesh width of the entire midgut BL is not completely uniform—neither in midguts of sugar-fed nor in those of (protein) meal-fed mosquitoes. Occasionally, even a midgut from a sugar-fed mosquito may contain zones were the BL mesh size is somewhat enlarged. In a few instances, SEM images showed slight tears in the BL surrounding the midgut of sugar-fed mosquitoes (see Figure S1; [21]. Our data also demonstrate that the number of midguts obtained from proteinmeal-fed mosquitoes showing association with 50 nm gold-nanoparticles strongly fluctuated between the various time points. In conjunction with our explanation above, we speculate that meal ingestion into the midgut may not uniformly expand the mesh width of the entire BL surrounding each midgut. Previously, we suggested that following bloodmeal digestion, the midgut BL structure would reconstitute to its pre-bloodmeal condition with the help of extracellular proteases such as matrix-metalloproteinases (MMPs) [25,44]. Our data here suggest that, similar to observations made by Reinhardt and Hecker [29], the mesh width of the midgut BL remains distended for a prolonged period of time after bloodmeal digestion. We hypothesize that this is a critical phenomenon enabling ZIK virions to disseminate from the midgut after complete digestion of the bloodmeal. Our experiments did not confirm whether the midgut BL structure remains permanently altered after a single bloodmeal ingestion since our observation period did not exceed 168 h pf.

Previous reports have shown that DENV2 titers were significantly increased in mosquitoes, which had ingested a subsequent, non-infectious bloodmeal several days after ingestion of an initial, DENV2-containing bloodmeal [45,46]. Our ultrastructural data here show that DENV4 accumulation at the epithelial side of the midgut BL was strongly increased at 144 h pibm in those females that had acquired a second, non-infectious bloodmeal 120 h after the initial, DENV4 containing bloodmeal. Thus, the enhancement effect of subsequent bloodmeal ingestion was apparent before the virus exited the midgut. This leads us to conclude that ingestion of the second, non-infectious bloodmeal might provide a nutritional boost for the already virus-infected midgut epithelium thereby increasing virus production.

## 5. Conclusions

In summary, our study suggests that the initial bloodmeal ingested by a female mosquito is priming the midgut structure including its BL to become permissive for ZIK virions. We also show that following a single virus containing bloodmeal, the midgut BL maintains for at least 120 h a modified, distended structure enabling flaviviruses such as ZIKV to disseminate from the midgut after bloodmeal digestion. A subsequent non-infectious bloodmeal acquired after the initial virus containing bloodmeal, as shown for DENV4, leads to strongly increased virus production in the midgut epithelium.

## Figures and Tables

**Table 1:** Number of dissected midguts obtained at various time points post-feeding from sugar-fed or proteinmeal-fed females showing FITC and/or Cy3 signaling following 2 h soaking in a 5 nm (FITC labeled)/50 nm (Cy3 labeled) gold-nanoparticle suspension.

		24 h pf ^1^	48 h pf	54 h pf	60 h pf	72 h pf	96 h pf	120 h pf	168 h pf
Proteinmeal	FITC positive	34/34 ^2^	26/26	14/14	25/25	22/22	12/12	9/9	8/8
		100%	100%	100%	100%	100%	100%	100%	100%
	Cy3 positive	9/34	4/26	3/14	11/25	7/22	3/12	2/9	0/8
		26.5%	15.4%	21.4%	44.0%	31.8%	25.0%	22.2%	0
Sugarmeal	FITC positive	22/22	18/18	6/6	17/17	20/20	18/18	13/13	20/20
		100%	100%	100%	100%	100%	100%	100%	100%
	Cy3 positive	1/22	1/18	0/6	2/17	0/20	2/18	1/13	2/20
		4.5%	5.6%	0	11.8%	0	11.1%	7.7%	10.0%

^1^ pf: post-feeding. ^2^ number of positive midguts divided by total number of midguts examined.

## Supplementary Materials

The following are available online at https://www.mdpi.com/1999-4915/11/11/1056/s1, Figure S1. Ultrastructural imaging of the midgut surface of sugar-fed and proteinmeal-fed *Ae. aegypti* by scanning electron microscopy (SEM). The surface of sugar-fed midguts occasionally showed slight tears. Overall surface damage was much more pronounced among those midguts, which were obtained from females at 24 h pf of a proteinmeal consisting of a 20% BSA solution. By this time point, the midgut tissue was strongly distended due to the presence of the proteinmeal. At 168 h pf, the proteinmeal was completely digested and the surface of the midgut tissue appeared relaxed with occasional minor damage resembling that of sugar-fed midguts. Images were acquired using a FEI Quanta 600F environmental scanning electron microscope (FEI, Hillsboro, OR, USA); Figure S2: Variability in signal detection for 5 nm and 50 nm gold-nanoparticles among individual midguts, which had been dissected from female mosquitoes at 60 h pf of a proteinmeal (20% BSA solution). Dissected midguts were soaked for 2 h in a suspension of 5 nm (labeled with FITC, 1.99 × 10^9^ nanoparticles/mL) and 50 nm (labeled with Cy3, 1.99 × 10^9^ nanoparticles/mL) gold-nanoparticles before mounting and viewing. Midgut samples were viewed under a confocal microscope thereby maintaining the same imaging parameters and microscope settings for each sample. Images were captured using an inverted spectral confocal microscope (TCP SP8 MP, Leica Microsystems, Wetzlar, Germany).

## References

[1] Dick G.W., Kitchen S.F., Haddow A.J. (1952). Zika virus. I. Isolations and serological specificity. Trans. R. Soc. Trop. Med. Hyg..

[2] Zanluca C., Melo V.C., Mosimann A.L., Santos G.I., Santos C.N., Luz K. (2015). First report of autochthonous transmission of Zika virus in Brazil. Mem. Inst. Oswaldo. Cruz..

[3] Fauci A.S., Morens D.M. (2016). Zika virus in the Americas-yet another arbovirus threat. N. Engl. J. Med..

[4] Zhang Q., Sun K., Chinazzi M., Pastore Y.P.A., Dean N.E., Rojas D.P., Merler S., Mistry D., Poletti P., Rossi L. (2017). Spread of Zika virus in the Americas. Proc. Natl. Acad. Sci. USA.

[5] Musso D., Gubler D.J. (2016). Zika virus. Clin. Microbiol. Rev..

[6] Broutet N., Krauer F., Riesen M., Khalakdina A., Almiron M., Aldighieri S., Espinal M., Low N., Dye C. (2016). Zika Vvrus as a cause of neurologic disorders. N. Engl. J. Med..

[7] Cauchemez S., Besnard M., Bompard P., Dub T., Guillemette-Artur P., Eyrolle-Guignot D., Salje H., Van Kerkhove M.D., Abadie V., Garel C. (2016). Association between Zika virus and microcephaly in French Polynesia, 2013–2015: A retrospective study. Lancet.

[8] Mlakar J., Korva M., Tul N., Popovic M., Poljsak-Prijatelj M., Mraz J., Kolenc M., Resman Rus K., Vesnaver Vipotnik T., Fabjan Vodusek V. (2016). Zika virus associated with microcephaly. N. Engl. J. Med..

[9] Chouin-Carneiro T., Vega-Rua A., Vazeille M., Yebakima A., Girod R., Goindin D., Dupont-Rouzeyrol M., Lourenco-de-Oliveira R., Failloux A.B. (2016). Differential susceptibilities of *Aedes aegypti* and *Aedes albopictus* from the Americas to Zika virus. PLoS Negl. Trop. Dis..

[10] Guerbois M., Fernandez-Salas I., Azar S.R., Danis-Lozano R., Alpuche-Aranda C.M., Leal G., Garcia-Malo I.R., Diaz-Gonzalez E.E., Casas-Martinez M., Rossi S.L. (2016). Outbreak of Zika virus infection, Chiapas State, Mexico, 2015, and First confirmed transmission by *Aedes aegypti* mosquitoes in the Americas. J. Infect. Dis..

[11] Marcondes C.B., Ximenes Mde F. (2016). Zika virus in Brazil and the danger of infestation by *Aedes* (Stegomyia) mosquitoes. Rev. Soc. Bras. Med. Trop..

[12] Weger-Lucarelli J., Ruckert C., Chotiwan N., Nguyen C., Garcia Luna S.M., Fauver J.R., Foy B.D., Perera R., Black W.C., Kading R.C. (2016). Vector competence of American mosquitoes for three strains of Zika virus. PLoS Negl. Trop. Dis..

[13] Hardy J.L., Houk E.J., Kramer L.D., Reeves W.C. (1983). Intrinsic factors affecting vector competence of mosquitoes for arboviruses. Annu. Rev. Entomol..

[14] Franz A.W., Kantor A.M., Passarelli A.L., Clem R.J. (2015). Tissue barriers to arbovirus infection in mosquitoes. Viruses.

[15] Junjhon J., Pennington J.G., Edwards T.J., Perera R., Lanman J., Kuhn R.J. (2014). Ultrastructural characterization and three-dimensional architecture of replication sites in dengue virus-infected mosquito cells. J. Virol..

[16] Plevka P., Battisti A.J., Sheng J., Rossmann M.G. (2014). Mechanism for maturation-related reorganization of flavivirus glycoproteins. J. Struct. Biol..

[17] Chambers T.J., Hahn C.S., Galler R., Rice C.M. (1990). Flavivirus genome organization, expression, and replication. Annu. Rev. Microbiol..

[18] Kuhn R.J., Zhang W., Rossmann M.G., Pletnev S.V., Corver J., Lenches E., Jones C.T., Mukhopadhyay S., Chipman P.R., Strauss E.G. (2002). Structure of dengue virus: Implications for flavivirus organization, maturation, and fusion. Cell.

[19] Natori Y., O’Meara Y.M., Manning E.C., Minto A.W., Levine J.S., Weise W.J., Salant D.J. (1992). Production and polarized secretion of basement membrane components by glomerular epithelial cells. Am. J. Physiol..

[20] Yurchenco P.D., O’Rear J.J. (1994). Basal lamina assembly. Curr. Opin. Cell Biol..

[21] Kantor A.M., Grant D.G., Balaraman V., White T.A., Franz A.W.E. (2018). Ultrastructural analysis of chikungunya virus dissemination from the midgut of the yellow fever mosquito, *Aedes aegypti*. Viruses.

[22] Girard Y.A., Popov V., Wen J., Han V., Higgs S. (2005). Ultrastructural study of West Nile virus pathogenesis in *Culex pipiens quinquefasciatus* (Diptera: Culicidae). J. Med. Entomol..

[23] Whitfield S.G., Murphy F.A., Sudia W.D. St. (1973). Louis encephalitis virus: An ultrastructural study of infection in a mosquito vector. Virology.

[24] Salazar M.I., Richardson J.H., Sanchez-Vargas I., Olson K.E., Beaty B.J. (2007). Dengue virus type 2: Replication and tropisms in orally infected *Aedes aegypti* mosquitoes. BMC Microbiol..

[25] Dong S., Balaraman V., Kantor A.M., Lin J., Grant D.G., Held N.L., Franz A.W.E. (2017). Chikungunya virus dissemination from the midgut of *Aedes aegypti* is associated with temporal basal lamina degradation during bloodmeal digestion. PLoS Negl. Trop. Dis..

[26] Okuda K., de Souza Caroci A., Ribolla P.E., de Bianchi A.G., Bijovsky A.T. (2002). Functional morphology of adult female *Culex quinquefasciatus* midgut during blood digestion. Tissue Cell..

[27] Rudin W., Hecker H. (1979). Functional morphology of the midgut of *Aedes aegypti* L. (Insecta, Diptera) during blood digestion. Cell Tissue Res..

[28] Hynes R.O., Zhao Q. (2000). The evolution of cell adhesion. J. Cell Biol..

[29] Reinhardt C., Hecker H. (1973). Structure and function of the basal lamina and of the cell junctions in the midgut epithelium (stomach) of female *Aedes aegypti* L. (Insecta, Diptera). Acta. Trop..

[30] Jose J., Snyder J.E., Kuhn R.J. (2009). A structural and functional perspective of alphavirus replication and assembly. Future Microbiol..

[31] Solignat M., Gay B., Higgs S., Briant L., Devaux C. (2009). Replication cycle of chikungunya: A re-emerging arbovirus. Virology.

[32] Hernandez R., Brown D.T., Paredes A. (2014). Structural differences observed in arboviruses of the alphavirus and flavivirus genera. Adv. Virol..

[33] Apte-Sengupta S., Sirohi D., Kuhn R.J. (2014). Coupling of replication and assembly in flaviviruses. Curr. Opin. Virol..

[34] Silva L.A., Dermody T.S. (2017). Chikungunya virus: Epidemiology, replication, disease mechanisms, and prospective intervention strategies. J. Clin. Invest..

[35] Strauss J.H., Strauss E.G. (1994). The alphaviruses: Gene expression, replication, and evolution. Microbiol Rev..

[36] Jose J., Taylor A.B., Kuhn R.J. (2017). Spatial and temporal analysis of alphavirus replication and assembly in mammalian and mosquito cells. MBio.

[37] Scott T.W., Hildreth S.W., Beaty B.J. (1984). The distribution and development of Eastern equine encephalitis virus in its enzootic mosquito vector, Culiseta melanura. Am. J. Trop. Med. Hyg..

[38] Dong S., Kantor A.M., Lin J., Passarelli A.L., Clem R.J., Franz A.W. (2016). Infection pattern and transmission potential of chikungunya virus in two New World laboratory-adapted *Aedes aegypti* strains. Sci. Rep..

[39] Wendell M.D., Wilson T.G., Higgs S., Black W.C. (2000). Chemical and gamma-ray mutagenesis of the white gene in *Aedes aegypti*. Insect Mol. Biol..

[40] Sheridan M.A., Balaraman V., Schust D.J., Ezashi T., Roberts R.M., Franz A.W.E. (2018). African and Asian strains of Zika virus differ in their ability to infect and lyse primitive human placental trophoblast. PLoS ONE.

[41] Hammon W.M., Rudnick A., Sather G., Rogers K.D., Morse L.J. (1960). New hemorrhagic fevers of children in the Philippines and Thailand. Trans. Assoc. Am. Physicians..

[42] Hammon W.M., Rudnick A., Sather G.E. (1960). Viruses associated with epidemic hemorrhagic fevers of the Philippines and Thailand. Science.

[43] Houk E.J., Hardy J.L., Chiles R.E. (1981). Permeability of the midgut basal lamina in the mosquito, *Culex tarsalis* Coquillett (Insecta, Diptera). Acta. Trop..

[44] Kantor A.M., Dong S., Held N.L., Ishimwe E., Passarelli A.L., Clem R.J., Franz A.W. (2017). Identification and initial characterization of matrix metalloproteinases in the yellow fever mosquito, *Aedes aegypti*. Insect Mol. Biol..

[45] Molina-Cruz A., Gupta L., Richardson J., Bennett K., Black W., Barillas-Mury C. (2005). Effect of mosquito midgut trypsin activity on dengue-2 virus infection and dissemination in *Aedes aegypti*. Am. J. Trop. Med. Hyg..

[46] Sanchez-Vargas I., Harrington L.C., Doty J.B., Black W.C., Olson K.E. (2018). Demonstration of efficient vertical and venereal transmission of dengue virus type-2 in a genetically diverse laboratory strain of *Aedes aegypti*. PLoS Negl. Trop. Dis..

