# Supplementary figures and images for: Zika Virus Dissemination from the Midgut of Aedes aegypti is Facilitated by Bloodmeal-Mediated Structural Modification of the Midgut Basal Lamina
Source: Viruses. 2019 Nov 14;11(11):1056. doi: 10.3390/v11111056 (PMC6893695; doi:10.3390/v11111056)

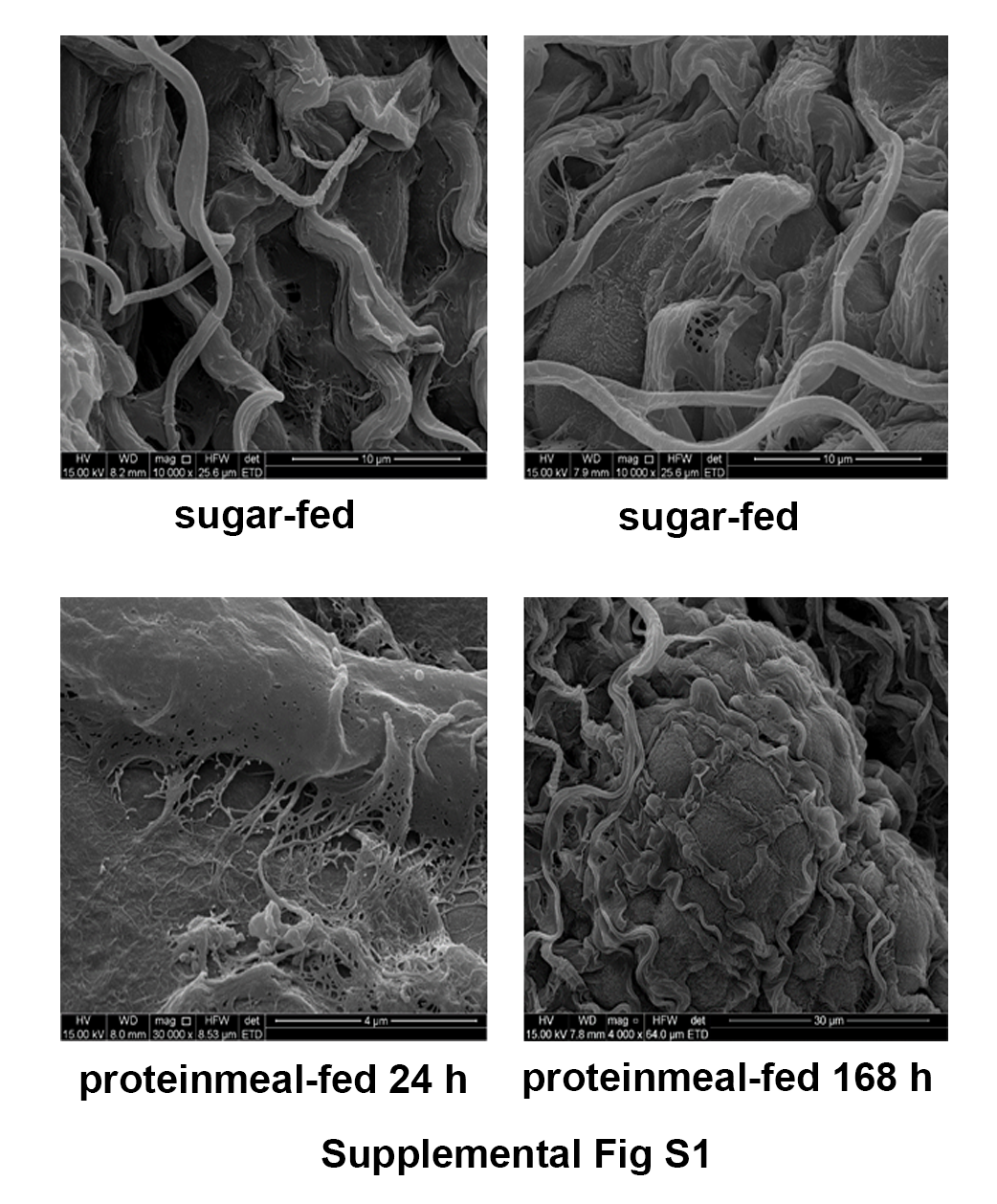

Supplement: Supplementary file 1 [file viruses-11-01056-s001.zip › supp/Supplemental Fig 1.tif]

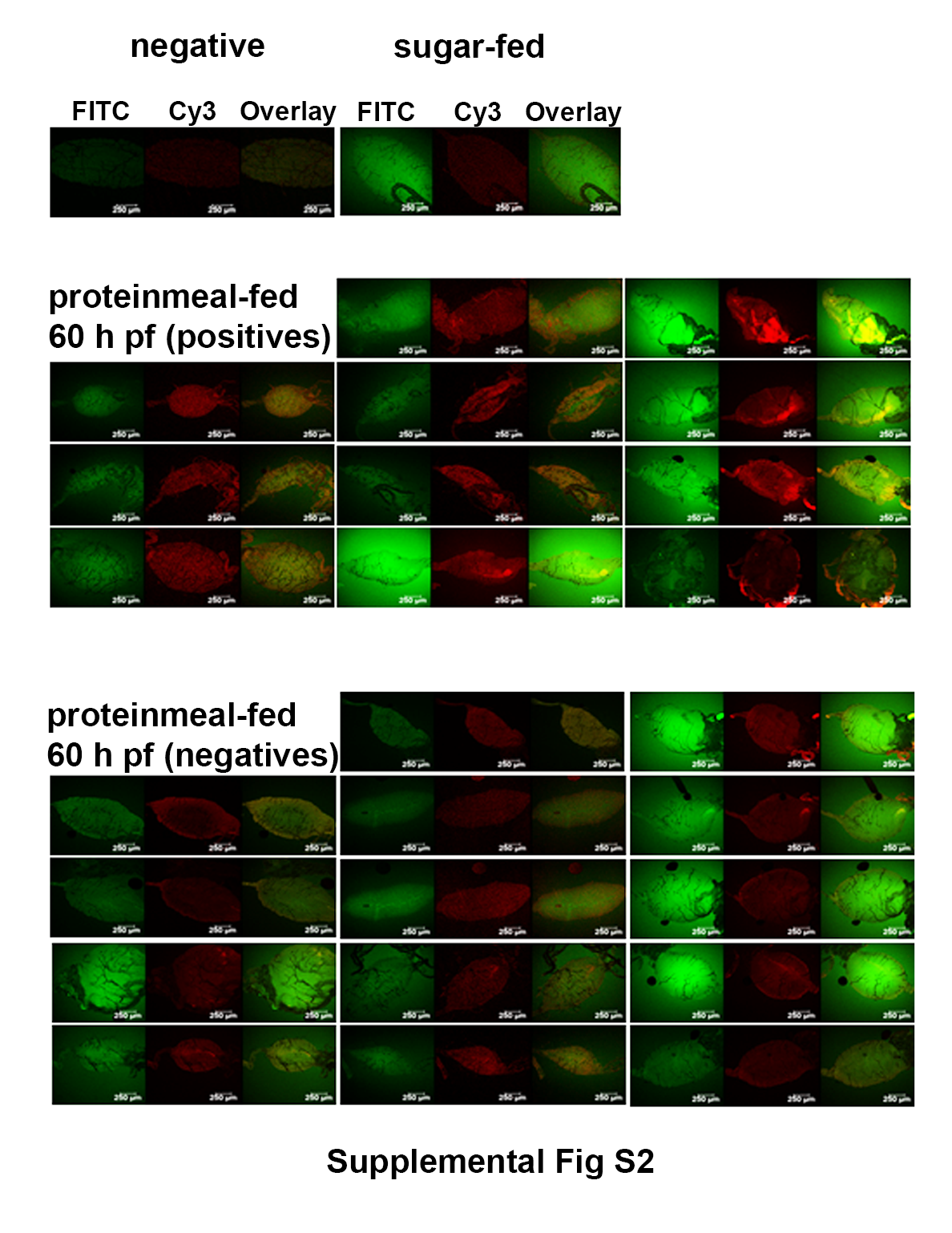

Supplement: Supplementary file 1 [file viruses-11-01056-s001.zip › supp/Supplemental Fig 2.tif]
